# Intestinal fatty acid-binding protein, a biomarker of intestinal barrier dysfunction, increases with the progression of type 2 diabetes

**DOI:** 10.7717/peerj.10800

**Published:** 2021-02-02

**Authors:** Yifeng Wang, Licheng Ding, Jiayue Yang, Lijun Liu, Liang Dong

**Affiliations:** 1 Department of Emergency and Critical Care Medicine, Second Affiliated Hospital of Soochow University, Suzhou, China; 2 Department of Critical Care Medicine, Wuxi People’s Hospital affiliated to Nanjing Medical University, Wuxi, China; 3 Department of Emergency Medicine, Affiliated Hospital of Jiangnan University, Wuxi, China; 4 Department of Endocrinology and Metabolism, Wuxi People’s Hospital affiliated to Nanjing Medical University, Wuxi, China; 5 Department of Critical Care Medicine, Taizhou Central Hospital (Taizhou University Hospital), Taizhou, China

**Keywords:** Intestinal fatty acid-binding protein (I-FABP), Intestinal barrier (IB) dysfunction, Hyperglycemia, Progression of diabetes

## Abstract

**Objective:**

To investigate serum intestinal fatty acid-binding protein (I-FABP) in two groups of patients with different duration of hyperglycemia in a cross-sectional study.

**Materials and Methods:**

In the present study, a total of 280 individuals (158 outpatients and 122 inpatients) suffering from hyperglycemia were recruited between May and September 2019. The clinical information of all participants was collected from the hospital information system, including the duration of hyperglycemia, age, gender, hemoglobin A1c (HbA1c), 75-g oral glucose tolerance test including fasting plasma glucose (FPG), 2-hour plasma glucose (2hPG), fasting C-peptide (FC-pep), 2-hour C-peptide (2hC-pep), fasting insulin (FIns), and 2-hour insulin (2hIns). In addition, the morbidity of diabetic complications (retinopathy, neuropathy, and nephropathy) in the inpatient group was determined. Furthermore, the difference between 2hPG and FPG (ΔPG), the difference between 2hC-pep and FC-pep (ΔC-pep), and the difference between 2hIns and FIns (ΔIns) were calculated. The level of serum I-FABP, a biomarker of intestinal barrier (IB) dysfunction, was estimated by an enzyme-linked immunosorbent assay.

**Results:**

For the outpatient group, the median duration of hyperglycemia was less than a year; the serum I-FABP level was positively correlated with age (*R* = 0.299, *P* < 0.001). For the inpatient group, the median duration of hyperglycemia was ten years; correlation analysis showed that the serum I-FABP level was positively associated with age and ΔPG (*R* = 0.286, *P* = 0.001; *R* = 0.250, *P* = 0.006, respectively) while negatively associated with FC-pep and 2hC-pep (*R* =  − 0.304, *P* = 0.001; *R* =  − 0.241, *P* = 0.008, respectively); multiple linear regression analysis showed that the serum I-FABP level was positively associated with the duration of hyperglycemia (β = 0.362, *P* < 0.001); moreover, patients with retinopathy had a significantly higher I-FABP level than those without retinopathy (*P* = 0.001).

**Conclusions:**

In the outpatients whose duration of hyperglycemia was less than a year, the serum I-FABP level was positively associated with age. In the inpatients with different courses of diabetes, the serum I-FABP level was positively associated with the duration of hyperglycemia and glycemic variability but negatively associated with islet beta-cell function; moreover, the serum I-FABP level was higher in patients with retinopathy than in those without retinopathy, suggesting that the IB dysfunction got worse with the progression of diabetes.

## Introduction

Diabetes has become a global public health issue with a growing morbidity and financial burden in the past few decades. Recently, diabetes has been considered as a risk factor of a worse prognosis in patients with COVID-19 (Zhu et al., 2020). Interestingly, as the most typical clinical feature of diabetes, hyperglycemia has been confirmed to be an essential predictor of adverse outcomes in diabetic as well as in nondiabetic patients in a previous study (Cichosz & Schaarup, 2017). The association between hyperglycemia and adverse outcomes in nondiabetic patients is apparent because reversible hyperglycemia, also known as stress-induced hyperglycemia (SIH), is a secondary symptom of primary disease and a well-known marker of disease severity (Mifsud, Schembri & Gruppetta, 2018). However, since hyperglycemia exists long before other diseases appear in diabetic patients, the relationship between preexisting hyperglycemia and adverse outcomes needs to be investigated.

Adverse outcomes associated with diabetes can partly be explained by the vulnerablity of diabetic patients to systemic infection and inflammatory response (Knapp, 2013). Nowadays, intestinal barrier (IB) dysfunction induced by hyperglycemia (Thaiss et al., 2018) is considered to be the underlying mechanism of systemic infection and inflammatory response in diabetic patients. The IB is the interface between the gut microbiome and the human body, it prevents the gut microbiome and other deleterious intestinal contents from crossing the barrier during enteral nutrition absorption. In addition, IB dysfunction results in the translocation of intestinal contents, which could be the direct cause of systemic infection and inflammatory response (Camilleri, 2019). In a study by Thaiss et al. (2018), hyperglycemia was confirmed to be an independent risk factor for IB dysfunction in animals; furthermore, the association between hyperglycemia and IB dysfunction was observed to be time-dependent and dose-dependent in vitro. Although the relationship between hyperglycemia and IB dysfunction has been clarified in animals and cell-based models (Thaiss et al., 2018), the clinical evidence in humans is lacking.

In this study, the serum concentration of intestinal fatty acid-binding protein (I-FABP) was used to indicate the severity of IB dysfunction. I-FABP is an intracellular protein specifically and abundantly expressed in intestinal epithelial cells, and its increased serum concentration represents intestinal epithelial cell damage and IB dysfunction (Gajda & Storch, 2015; Wells et al., 2017). As a biomarker of IB dysfunction, I-FABP determination has been used in patients with necrotizing enterocolitis (Coufal et al., 2020), acute mesenteric ischemia (Salim et al., 2017), strangulated small bowel obstruction (Kittaka et al., 2014), Crohn’s disease (Sarikaya et al., 2015), blunt trauma (Matsumoto et al., 2017), celiac disease (Oldenburger et al., 2018), acute pancreatitis(Kupcinskas et al., 2018; Goswami et al., 2017), acute decompensated heart failure (Kitai et al., 2017), chronic renal failure (Okada et al., 2018), septic shock (Sekino et al., 2017), psoriasis (Sikora et al., 2019), and even physiological stressor-induced intestinal damage (March et al., 2017). In addition to serum I-FABP, many other biomarkers are used to measure IB function (Wells et al., 2017; Piton & Capellier, 2016). However, in this study, the serum I-FABP level was determined to evaluate IB dysfunction as it is convenient to measure in a noninvasive manner.

Diabetic and prediabetic patients with different severities and durations of hyperglycemia were recruited, and their serum I-FABP levels were measured in this study.

## Materials and Methods

### Study participants

In this cross-sectional study, participants were recruited from the outpatient clinic and the inpatient ward of the Department of Endocrinology and Metabolism, Wuxi People’s Hospital affiliated to Nanjing Medical University. The outpatients were included in this study if they had been tested to be hyperglycemic or had diabetes-related symptoms within a year without taking antidiabetic drugs. The inpatients were included in this study if they had been diagnosed with diabetes and were newly admitted to the inpatient ward. However, participants were excluded from this study if they were younger than 18 years old; had SIH, type 1 diabetes, or a change in lifestyle (e.g., diet or exercise) in the past year; were pregnant in the past year; or had acute complications of diabetes, severe hepatic, renal, or heart insufficiency, acute digestive system disease, or abdominal surgery in the past half year or acute infection in the past month. All patients who agreed to this study and met the inclusion criteria were included in the analysis. Most conditions causing an increase in the serum I-FABP level were excluded according to the criteria, which might have caused a selection bias. Patients who suffered from complications of severe nephropathy were not included in this study. They were admitted to the Nephrology Department rather than the Department of Endocrinology and Metabolism, which resulted in a selection bias.

The present study was approved by the Research Ethics Committee of Wuxi People’s Hospital affiliated to Nanjing Medical University (HS2019003). Each participant signed an informed consent form. This study was registered at the Chinese Clinical Trial Register Center (ChiCTR1900022026) and was carried out from May to September 2019. All data can be obtained from the corresponding authors on demand.

### Clinical information

The clinical information of all participants was collected from the hospital information system, including the duration of hyperglycemia, age, gender, hemoglobin A1c (HbA1c), 75-g oral glucose tolerance test including fasting plasma glucose (FPG), 2-hour plasma glucose (2hPG), fasting C-peptide (FC-pep), 2-hour C-peptide (2hC-pep), fasting insulin (FIns), and 2-hour insulin (2hIns). In addition, the morbidity of diabetic complications (retinopathy, neuropathy, and nephropathy) in the inpatient group was determined. Furthermore, the difference between 2hPG and FPG (ΔPG), the difference between 2hC-pep and FC-pep (ΔC-pep), and the difference between 2hIns and FIns (ΔIns) were calculated.

### Serum I-FABP estimation

All of the fasting blood samples collected from the patients to perform the necessary tests were stored at 2−8 °C in the clinical laboratory of Wuxi People’s Hospital. Then certain serum samples of the enrolled patients were aliquoted and stored at −80 °C within 8 h for future research. The serum concentration of I-FABP was estimated in duplicate by enzyme-linked immunosorbent assay (R&D, USA and Canada, Catalog Number DFBP20) according to the standardized protocol, and the estimated mean values were used for further analysis.

### Statistical analysis

Statistical analysis was assessed using SPSS 25 (IBM Corporation, Chicago, IL, USA). Continuous variables were assessed for normality using the Kolmogorov–Smirnov test. Variables with a non-normal distribution were summarized by the median and interquartile range. Differences between two groups of continuous variables with non-normal distributions were assessed by the Mann–Whitney test. Differences between categorical variables were assessed by the chi-squared test. The relationships between two continuous variables were assessed by the Spearman correlation coefficient. Logarithmic transformation was performed when necessarily. The associations showing a *P*-value <0.05 in the correlation analysis were included in the multiple linear regression analysis. Only those metrics with a *P*-value <0.05 were kept in the final multiple linear regression models with stepwise variable selection. Finally, all of the differences were considered significant at a 5% level.

## Results

### Serum I-FABP in the outpatient group

The outpatients were divided into prediabetes group and diabetes group according to the latest diagnosis standards for diabetes (American Diabetes Association, 2020). The clinical information and serum I-FABP levels of these two groups are tabulated in Table 1. Compared with the prediabetes group, the diabetes group had a higher HbA1c, FPG, FC-pep, 2hPG, and ΔPG but a lower 2hC-pep, 2hIns, ΔC-pep, and ΔIns. However, there were no statistically significant differences in age, FIns, or serum I-FABP between these two groups.

**Table 1:** Clinical information and the serum I-FABP level between the different stages of diabetes for the outpatient group.

	Prediabetes	Diabetes	*P*-value
Male/Female, n^**^	42/53	48/15	<0.001
Age, y	45 (32, 57)	49 (34, 55)	0.790
HbA1c, %^**^	5.5 (5.3, 5.8)	6.9 (6.4, 9.1)	<0.001
FPG, mmol/L^**^	5.94 (5.41, 6.47)	8.54 (7.64, 10.89)	<0.001
FC-pep, ng/mL^**^	2.39 (1.86, 3.16)	2.97 (2.29, 3.62)	0.004
FIns, mu/L	10.21 (5.91, 14.36)	11.98 (7.37, 15.13)	0.136
2hPG, mmol/L^**^	8.46 (6.93, 10.28)	15.73 (13.55, 19.25)	<0.001
2hC-pep, ng/mL^**^	9.96 (7.61, 12.83)	7.35 (5.89, 11.15)	0.001
2hIns, mu/L^**^	64.26 (42.18, 122.03)	46.56 (28.78, 69.34)	0.003
ΔPG, mmol/L^**^	2.52 (1.00, 4.00)	7.28 (4.81, 9.21)	<0.001
ΔC-pep, ng/mL^**^	7.56 (5.57, 10.36)	4.63 (3.03, 7.96)	<0.001
ΔIns, mu/L^**^	57.25 (33.78, 105.98)	34.82 (18.20, 58.76)	<0.001
I-FABP, pg/mL	1354 (948, 1634)	1231 (883, 1604)	0.442

**Notes.**

I-FABP intestinal fatty acid-binding protein HbA1c hemoglobin A1c FPG fasting plasma glucose FC-pep fasting C-peptide FIns fasting insulin 2hPG 2-hour plasma glucose 2hC-pep 2-hour C-peptide 2hIns 2-hour insulin ΔPG 2hPG –FPG ΔC-pep 2hC-pep –FC-pep ΔIns 2hIns –FIns

** *P* < 0.01.

Furthermore, the correlation analysis of the serum I-FABP level was compared with age, HbA1c, FPG, FC-pep, FIns, 2hPG, 2hC-pep, 2hIns, ΔPG, ΔC-pep, and ΔIns. However, age was the only metric that was statistically correlated with the serum I-FABP level in the outpatient group (Table 2).

**Table 2:** Correlation analysis of the serum I-FABP level for the outpatient group.

		R	*P*-value
I-FABP	Age^**^	0.299	<0.001
	HbA1c	−0.027	0.734
	FPG	−0.052	0.515
	FC-pep	0.031	0.696
	FIns	0.010	0.899
	2hPG	−0.025	0.757
	2hC-pep	0.104	0.197
	2hIns	0.063	0.435
	ΔPG	0.003	0.973
	ΔC-pep	0.103	0.199
	ΔIns	0.080	0.322

**Notes.**

I-FABP intestinal fatty acid-binding protein HbA1c hemoglobin A1c FPG fasting plasma glucose FC-pep fasting C-peptide FIns fasting insulin 2hPG 2-hour plasma glucose 2hC-pep 2-hour C-peptide 2hIns 2-hour insulin ΔPG 2hPG –FPG ΔC-pep 2hC-pep –FC-pep ΔIns 2hIns –FIns

** *P* < 0.01.

### Serum I-FABP in the inpatient group

The correlation analysis for the inpatient group showed that the serum I-FABP level was significantly correlated with age, duration of hyperglycemia, FC-pep, 2hC-pep, and ΔPG but not significantly correlated with the other metrics. However, the duration of hyperglycemia was the only metric that was statistically associated with the serum I-FABP level in the multiple linear regression analysis (Table 3).

**Table 3:** Correlation analysis and multiple linear regression analysis of the serum I-FABP level for the inpatient group.

	Correlation analysis		Multiple linear regression	
	R	*P*-value	β	*P*-value
Age	0.286	0.001	0.098	0.317
Duration	0.350	<0.001	0.362	<0.001
HbA1c	0.045	0.625	–	–
FPG	−0.126	0.168	–	–
FC-pep	−0.304	0.001	−0.090	0.352
FIns	−0.145	0.244	–	–
2hPG	0.149	0.105	–	–
2hC-pep	−0.241	0.008	−0.064	0.494
2hIns	−0.219	0.081	–	–
ΔPG	0.250	0.006	0.149	0.110
ΔC-pep	−0.163	0.076	–	–
ΔIns	−0.209	0.097	–	–

**Notes.**

The corresponding model was *Y* = 7.292 + 0.027 * X, Y: ln(I-FABP), X: Duration, and *R*^2^ = 0.131 in the multiple linear regression.

I-FABP intestinal fatty acid-binding protein HbA1c hemoglobin A1c FPG fasting plasma glucose FC-pep fasting C-peptide FIns fasting insulin 2hPG 2-hour plasma glucose 2hC-pep 2-hour C-peptide 2hIns 2-hour insulin ΔPG 2hPG –FPG ΔC-pep 2hC-pep –FC-pep ΔIns 2hIns –FIns β standardized β-coefficient

Finally, we investigated the relationship between the serum I-FABP level and diabetic complications (retinopathy, neuropathy, and nephropathy). The inpatients were divided into complication-positive group and complication-negative group, and the differences of the serum I-FABP level between the complication-positive group and the complication-negative group were estimated. The results showed that the retinopathy-positive group had a higher serum I-FABP level than the retinopathy-negative group, while the differences of the serum I-FABP level for the two other complications were not statistically significant (Table 4).

**Table 4:** Relationship between the serum I-FABP level and diabetic complications for the inpatient group.

Complications	Positive cases (%)	I-FABP		
		Positive	Negative	*P*-value
Retinopathy^**^	48 (39.3)	2164 (1749, 3262)	1585 (1168, 2467)	0.001
Neuropathy	51 (41.8)	1949 (1373, 2953)	1697 (1169, 2617)	0.126
Nephropathy	34 (27.9)	1836 (1206, 2719)	1829 (1256, 2638)	0.833

**Notes.**

I-FABP intestinal fatty acid-binding protein

** *P* < 0.01.

### Serum I-FABP in all participants

The clinical information and serum I-FABP levels of all participants recruited from the outpatient clinic and the inpatient ward are tabulated in Table 5. The median duration of hyperglycemia was less than a year for the outpatients, while it was ten years for the inpatients. Moreover, compared with the outpatients, the inpatients had higher age, HbA1c, FPG, 2hPG, ΔPG, and I-FABP but lower FC-pep, FIns, 2hC-pep, 2hIns, ΔC-pep, and ΔIns.

**Table 5:** Clinical information and serum I-FABP levels of participants recruited from the outpatient clinic and the inpatient ward.

	Outpatient	Inpatient	*P*-value
Male/Female, n	90/68	72/50	0.730
Age, y^**^	47.5 (33, 56)	56 (51.75, 67)	<0.001
Duration, y^**^	<1	10 (3, 14)	<0.001
HbA1c, %**	5.9 (5.5, 6.5)	8.0 (6.8, 9.3)	<0.001
FPG, mmol/L^*^	6.57 (5.77, 8.04)	7.43 (5.86, 9.08)	0.037
FC-pep, ng/mL^**^	2.69 (1.92, 3.45)	1.92 (1.29, 2.67)	<0.001
FIns, mu/L^*^	10.66 (6.29, 14.74)	7.77 (5.21, 12.74)	0.020
2hPG, mmol/L^**^	10.55 (7.87, 14.71)	18.97 (14.37, 21.38)	<0.001
2hC-pep, ng/mL^**^	9.22 (7.03, 12.39)	5.19 (3.59, 7.69)	<0.001
2hIns, mu/L^**^	55.34 (35.06, 109.25)	38.11 (21.67, 69.28)	0.001
ΔPG, mmol/L^**^	3.80 (1.91, 7.16)	10.32 (6.71, 12.95)	<0.001
ΔC-pep, ng/mL^**^	6.78 (4.27, 9.36)	3.33 (2.13, 5.17)	<0.001
ΔIns, mu/L^**^	47.02 (26.69, 91.97)	30.51 (16.84, 56.10)	0.002
I-FABP, pg/mL^**^	1289 (909, 1629)	1831 (1243, 2642)	<0.001

**Notes.**

I-FABP intestinal fatty acid-binding protein HbA1c hemoglobin A1c FPG fasting plasma glucose FC-pep fasting C-peptide FIns fasting insulin 2hPG 2-hour plasma glucose 2hC-pep 2-hour C-peptide 2hIns 2-hour insulin ΔPG 2hPG –FPG ΔC-pep 2hC-pep –FC-pep ΔIns 2hIns –FIns

* *P* < 0.05.

** *P* < 0.01.

Next, correlation analysis of the serum I-FABP level showed a statistical significance with age, duration of hyperglycemia, HbA1c, FC-pep, 2hPG, 2hC-pep, ΔPG, and ΔC-pep. Furthermore, multivariate linear regression analysis showed that the serum I-FABP level was positively associated with the age and the duration of hyperglycemia in all participants (Table 6).

**Table 6:** Correlation analysis and multiple linear regression analysis of the serum I-FABP level for all participants.

	Correlation analysis		Multiple linear regression	
	R	*P*-value	β	*P*-value
Age	0.398	<0.001	0.248	<0.001
Duration	0.413	<0.001	0.311	<0.001
HbA1c	0.196	0.001	0.012	0.843
FPG	−0.019	0.755	–	–
FC-pep	−0.220	<0.001	0.030	0.609
FIns	−0.072	0.282	–	–
2hPG	0.250	<0.001	0.045	0.461
2hC-pep	−0.214	<0.001	0.018	0.763
2hIns	−0.075	0.265	–	–
ΔPG	0.311	<0.001	0.122	0.064
ΔC-pep	−0.171	0.004	0.012	0.843
ΔIns	−0.058	0.392	–	–

**Notes.**

The corresponding model was *Y* = 6.749 + 0.026 * X_1_ + 0.009 * X_2_, Y: ln(I-FABP), X_1_: Duration, X_2_: Age, and *R*^2^ = 0.232 in the multiple linear regression.

I-FABP intestinal fatty acid-binding protein HbA1c hemoglobin A1c FPG fasting plasma glucose FC-pep fasting C-peptide FIns fasting insulin 2hPG 2-hour plasma glucose 2hC-pep 2-hour C-peptide 2hIns 2-hour insulin ΔPG 2hPG –FPG ΔC-pep 2hC-pep –FC-pep ΔIns 2hIns –FIns β standardized β-coefficient

## Discussion

A previous study has confirmed the relationship between hyperglycemia and IB dysfunction in animals and cell-based models (Thaiss et al., 2018), and our study added evidence from humans to support this relationship. In the present study, we investigated the serum I-FABP levels in diabetic and prediabetic patients who suffered from different severities and durations of hyperglycemia. Our results showed that in the outpatients whose duration of hyperglycemia was less than a year, the serum I-FABP level was positively associated with age. In the inpatients with different courses of diabetes, the serum I-FABP level was positively associated with the duration of hyperglycemia and glycemic variability but negatively associated with islet beta-cell function; moreover, the serum I-FABP level was higher in patients with retinopathy than in those without retinopathy, suggesting that the IB dysfunction got worse with the progression of diabetes.

### IB dysfunction and severity of hyperglycemia: the serum I-FABP level was not associated with FPG, 2hPG, or HbA1c in outpatients

The study by Thaiss et al., (2018) confirmed the dose-dependent relationship between IB dysfunction and hyperglycemia in vitro. Since hyperglycemia in the outpatient group was just identified within a year and untreated, this group was suitable for investigation of the relationship between hyperglycemia severity and IB dysfunction. FPG and 2hPG show instantaneous plasma glucose (PG) levels, while HbA1c shows the average PG level in the past 2–3 months (American Diabetes Association, 2020). These three metrics were used to determine the severity of hyperglycemia in this study. Unfortunately, none of them showed a statistical correlation with the serum I-FABP level in the outpatient group, and correlation analysis of the inpatient group showed similar results.

These negative results might be explained by the lower severity of hyperglycemia in our clinical study compared with the in vitro study by Thaiss et al., (2018). In their in vitro study, IB dysfunction was observed at a glucose level of 8 g/L (about 44 mmol/L) (Thaiss et al., 2018), which is rarely achieved in clinical patients. In other words, the hyperglycemia in our study was not severe enough to cause a statistical difference in the serum I-FABP level. Therefore, a well-designed study in humans is needed in the future to answer the dose-dependent relationship between IB dysfunction and hyperglycemia.

### IB dysfunction and duration of hyperglycemia: the serum I-FABP level was associated with the duration of hyperglycemia in inpatients

After dose-dependent relationship discussed above, time-dependent relationship was assessed, the relationship between IB dysfunction and the duration of hyperglycemia was investigated in the study. The median duration of hyperglycemia was less than a year for the outpatient group, while it was ten years for the inpatient group. For the inpatient group, the serum I-FABP level was statistically associated with the duration of hyperglycemia both according to the correlation analysis and the multivariate linear regression analysis.

The relationship between IB dysfunction and the duration of hyperglycemia can be explained by the accumulation of advanced glycation end products (AGEs) caused by the long-standing hyperglycemic state in diabetes. AGEs have been demonstrated to contribute to diabetic complications in many studies, and the accumulation of AGEs and the activation of their receptors (RAGE) induce NADPH oxidase stimulation, reactive oxygen intermediate formation, nuclear factor- *κ*B activation, and gene transcription, which further lead to a sustained inflammatory reaction, cell damage, and organ dysfunction (Wautier, Guillausseau & Wautier, 2017). Surprisingly, both the accumulation of AGEs and the overexpression of RAGE were observed in the gastrointestinal tract of diabetic rats (Chen, Gregersen & Zhao, 2015), which contributes to IB dysfunction in diabetes.

### IB dysfunction and progression of diabetes: the serum I-FABP level was associated with retinopathy, glycemic variability and islet beta-cell dysfunction in inpatients

As a result of sustained hyperglycemia, diabetic complications develop with the progression of diabetes. In this study, the serum I-FABP level was higher in patients with retinopathy than in those without retinopathy in the inpatient group. As retinopathy is a complication that develops with the progression of diabetes, this result suggested that the IB dysfunction got worse with the progression of diabetes. Unfortunately, patients who suffered from severe nephropathy were not included in this study, this partly explained why the serum I-FABP level was not higher in the patients with neuropathy or nephropathy.

Previous studies have found that glycemic variability is closely related to diabetic complications. Glycemic variability consists of the magnitude of PG excursions and the frequency of the fluctuations. Compared with sustained hyperglycemia, glycemic variability has more deleterious effects in the pathogenesis of diabetic complications (Beck et al., 2019; Nusca et al., 2018). An observational study has found that type 2 diabetic patients incapable of maintaining stable PG levels were more likely to have IB injury (Shen et al., 2019), in other words, glycemic variability might be associated with IB dysfunction. In this study, ΔPG was used to indicate the glycemic variability, and the results showed that the serum I-FABP level was positively correlated with ΔPG in the inpatient group. Taken together, IB dysfunction was related to glycemic variability and was aggravated with the progression of diabetes. Since ΔPG is not enough to show the whole day glycemic variability, better designed studies are needed to further estimate the relationship between glycemic variability and IB dysfunction.

In addition, islet beta-cell dysfunction advances with the progression of diabetes (Bergman, Finegood & Kahn, 2002). In this study, the serum C-pep and insulin levels were used to indicate islet beta-cell function. The results showed that the serum I-FABP level was negatively associated with the C-pep (FC-pep and 2hC-pep) levels in the inpatient group. However, there was no statistical correlation between the serum I-FABP and insulin (FIns and 2hIns) levels. C-pep is secreted from beta cells at an equimolar concentration as insulin, however, the half-life of C-pep is longer than that of insulin, (Polonsky & Rubenstein, 1984) which makes C-pep a better metric for islet beta-cell function. In this study, the investigation of C-pep suggested that the aggravation of IB dysfunction was accompanied by the loss of islet beta-cell function in the progression of diabetes.

Although the serum I-FABP level was correlated with ΔPG, FC-pep, and 2hC-pep, these associations disappeared in the multivariate linear regression analysis. Given the roles of diabetic complications, glycemic variability, and advancing islet beta-cell dysfunction in the progression of diabetes, ΔPG, FC-pep, and 2hC-pep were considered to be confounding factors in the relationship between the serum I-FABP level and the duration of hyperglycemia. Since the duration of hyperglycemia closely followed the course of diabetes, this study suggested that the IB dysfunction got worse with the progression of diabetes.

### IB dysfunction and age: the serum I-FABP level was positively correlated with age in outpatients and inpatients

In the outpatient group, without the influence of the duration of hyperglycemia, the serum I-FABP level was positively correlated with age, suggesting that the older patients suffered from more severe IB dysfunction in the newly diagnosed diabetic and prediabetic patients. A previous study has found that IB dysfunction might be an important event in the aging process, and that is conserved across a broad range of species (Dambroise et al., 2016). Age-related loss of the heat-shock transcription factor has been confirmed to be involved in IB dysfunction by accelerating the decay of the intestinal subapical terminal web and impairing its interactions with cell junctions in *C. elegans* (Egge et al., 2019). The current study added evidence regarding the association between IB dysfunction and age in humans.

Similarly, in the inpatient group, the serum I-FABP level was positively correlated with age. However, the association between I-FABP and age disappeared in the multivariate linear regression analysis, leaving the duration of hyperglycemia as the only metric associated with the serum I-FABP level. Considering that the durations of hyperglycemia get longer when diabetic patients grow older, age might be a confounding factor in the relationship between the serum I-FABP level and the duration of hyperglycemia in the inpatient group.

### The different behaviors of two groups

At first glance, the results in all participants seem to be the combination of two groups: correlation analysis showed the serum I-FABP level was positively associated with the duration of hyperglycemia, age, and glycemic variability but negatively associated with islet beta-cell function; multivariate linear regression analysis showed that the serum I-FABP level was associated with both age and the duration of hyperglycemia.

When all patients were combined, there was some evidence of a relationship, but on further investigation, it turned out that there appeared to be different behaviors in each group, and the difference might be mainly caused by the significant difference in the duration of hyperglycemia. As a result, the relationship between I-FAPB and the other metrics reported for all participants might be driven by the group sizes. Consequently, the results for all participants need to be interpreted with caution.

Moreover, in all participants and in the inpatient group, the multivariate linear regression models were just ways of determining possible associations between I-FABP and the metrics, the direct causal relationship in humans needs further investigation.

### Perspective of the study

Based on the results of this study, we speculated that IB dysfunction occurred and developed along with the progression of diabetes. The relationship between hyperglycemia and IB dysfunction offers a new perspective on the clinical phenomenon of diabetes. On one hand, an impaired IB enhances the influx and systemic dissemination of intestinal bacteria, which explains the vulnerability of diabetic patients to infection (Trevelin et al., 2017). On the other hand, more intestinal contents and bacterial metabolites come across the impaired IB and are released into the blood circulation, causing systemic chronic oxidative stress and inflammatory response, which not only promote diabetic complications (Jha et al., 2018; Volpe et al., 2018) but also contribute directly to diabetes (McCracken, Monaghan & Sreenivasan, 2018).

The association between the duration of hyperglycemia and IB dysfunction was clarified in this study, but the underlying mechanisms have not been discussed. Previous studies showed that nondiabetic patients who suffered from SIH were more likely to get diabetes in the future (Kar et al., 2019; Plummer et al., 2016), which brought about a confusing hypothesis that reversible hyperglycemia could cause irreversible hyperglycemia. It is well recognized that some primary diseases like sepsis, trauma, burns, and surgery, which induce SIH (Mifsud, Schembri & Gruppetta, 2018), can directly cause IB dysfunction (Greis et al., 2017). Hyperglycemia-induced IB dysfunction allows for an enhanced influx of intestinal contents, and the induced infection and inflammatory response further intensify hyperglycemia (Mifsud, Schembri & Gruppetta, 2018). Surprisingly, IB dysfunction builds a bridge between SIH and diabetes, that is, IB dysfunction not only results from but also contributes to hyperglycemia. Since improving IB function by treating the primary disease has been shown to alleviate SIH (Mifsud, Schembri & Gruppetta, 2018), aiming to improve IB function might be a promising treatment strategy for diabetes.

Besides the new therapeutic approach for hyperglycemia, some widely used antidiabetic drugs have already shown protective potential for IB. Glucagon-like peptide 1, which controls meal-related glycemic excursions, alleviates gut inflammation and promotes the repairment of intestinal epithelial cells (Smits et al., 2016; Anbazhagan et al., 2017). Similar protective effects have also been observed with metformin (Rodriguez, Hiel & Delzenne, 2018) and berberine (Gong et al., 2017). Compared with the IB, the gut microbiome has drawn increasing attention in diabetes research. Although quite a few studies have shown that the gut microbiome influences the development of diabetes (Hills et al., 2019; Canfora et al., 2019), the underlying mechanism is still unclear. As the interface between the gut microbiome and diabetic patients, the IB is a promising candidate for mechanism research.

This study had some limitations. First, the participants recruited from the inpatient ward were admitted to the hospital for different pathological conditions, including but not limited to poor glucose control and diabetic complications. The influence of all these conditions on the serum I-FABP level was not thoroughly investigated. Second, the participants recruited from the inpatient ward had been treated with antidiabetic drugs, whether these drugs affected the serum I-FABP level was not investigated. Third, patients who suffered from severe nephropathy complications were not included in this study. Fourth, the results from all participants were driven by the group sizes because of the significant difference in the duration of hyperglycemia between groups.

## Conclusions

To the best of our knowledge, this study investigated the serum I-FABP level in diabetic and prediabetic patients for the first time, and the results showed that in the outpatients whose duration of hyperglycemia was less than a year, the serum I-FABP level was positively associated with age. In the inpatients with different courses of diabetes, the serum I-FABP level was positively associated with the duration of hyperglycemia and glycemic variability but negatively associated with islet beta-cell function; moreover, the serum I-FABP level was higher in patients with retinopathy than in those without retinopathy, suggesting that the IB dysfunction got worse with the progression of diabetes.

Considering the participation of the gut microbiome in the etiology of diabetes, it is difficult to ignore the role of IB, whose dysfunction might cause oxidative stress, systemic infection, and inflammatory response. By highlighting the IB in diabetes-related research, we offer a new perspective to interpret this familiar disease.

##  Supplemental Information

10.7717/peerj.10800/supp-1 Supplemental Information 1 Raw data.

10.7717/peerj.10800/supp-2 Supplemental Information 2 Raw data for diabetic complications.

## Abbreviations

I-FABP: intestinal fatty acid-binding protein
IB: intestinal barrier
HbA1c: hemoglobin A1c
OGTT: oral glucose tolerance test
PG: plasma glucose
C-pep: C-peptide
Ins: insulin
FPG: fasting plasma glucose
FC-pep: fasting C-peptide
FIns: fasting insulin
2hPG: 2-hour plasma glucose
2hC-pep: 2-hour C-peptide
2hIns: 2-hour insulin
PG: the difference between 2hPG and FPG
C-pep: the difference between 2hC-pep and FC-pep
Ins: the difference between 2hIns and FIns
SIH: stress-induced hyperglycemia
AGEs: advanced glycation end products
RAGE: AGEs receptor

## Additional Information and Declarations

### Data Availability

The following information was supplied regarding data availability: Raw measurements are available in the Supplemental Files.
